# Network-inference-based prediction of the COVID-19 epidemic outbreak in the Chinese province Hubei

**DOI:** 10.1007/s41109-020-00274-2

**Published:** 2020-07-08

**Authors:** Bastian Prasse, Massimo A. Achterberg, Long Ma, Piet Van Mieghem

**Affiliations:** Faculty of Electrical Engineering, Mathematics and Computer Science, Delft University of Technology, Delft, P.O Box 5031, 2600 GA The Netherlands

**Keywords:** Network inference, Epidemiology, COVID-19, Coronavirus, SIR model

## Abstract

At the moment of writing, the future evolution of the COVID-19 epidemic is unclear. Predictions of the further course of the epidemic are decisive to deploy targeted disease control measures. We consider a network-based model to describe the COVID-19 epidemic in the Hubei province. The network is composed of the cities in Hubei and their interactions (e.g., traffic flow). However, the precise interactions between cities is unknown and must be inferred from observing the epidemic. We propose the Network-Inference-Based Prediction Algorithm (NIPA) to forecast the future prevalence of the COVID-19 epidemic in every city. Our results indicate that NIPA is beneficial for an accurate forecast of the epidemic outbreak.

## Introduction

In December 2019, the novel coronavirus SARS-CoV-2 emerged in the Chinese city Wuhan (Munster et al. 2020). The SARS-CoV-2 virus causes the COVID-19 disease. Contrary to initial observations (Cheng and Shan 2020), the COVID-19 virus does spread from person to person, as confirmed in Chan et al. (2020). On March 19, 2020, there were more than 215,000 confirmed infections, and more than 8500 people died (World Health Organization 2020; ‘Situation Update Worldwide, as of 18 March 2020’, www.ecdc.europa.eu/en/geographical-distribution-2019-nCoV-cases, unpublished; ‘Coronavirus (COVID-19)’, www.cdc.gov/coronavirus/2019-nCoV/index.html, unpublished). Assessing the further spread of the COVID-19 epidemic poses a major public health concern.

Many studies aim to estimate the basic reproduction number *R*_0_ of the COVID-19 epidemic (Zhao et al. 2020; Majumder and Mandl 2020; Li et al. 2020; Yang et al. 2020; Imai et al. 2019; Liu et al. 2020; Riou and Althaus 2020; Read et al. 2020; Wu et al. 2020). The basic reproduction number *R*_0_ is a crucial quantity to evaluate the hostility of a virus (Hethcote 2000; Heesterbeek 2002). The basic reproduction number *R*_0_ is defined (Diekmann et al. 1990) as “The expected number of secondary cases produced, in a completely susceptible population, by a typical infective individual during its entire period of infectiousness”. The greater the basic reproduction *R*_0_, the more individuals are infected in the long-term endemic state of the virus. If *R*_0_<1, then the virus dies out. The estimates for the basic reproduction number *R*_0_ of the COVID-19 epidemic range from *R*_0_=2.0 to *R*_0_=3.77.

The basic reproduction number *R*_0_ only coarsely assesses the quantitative behaviour of the epidemic. To obtain a more detailed picture of the epidemic, the development of epidemic outbreak prediction methods is focal. A diverse body of research considers the prediction of general epidemics. For instance, prediction methods are based on Kalman filtering (Yang et al. 2014), Bayesian model averaging (Yamana et al. 2017), basic regression (Brooks et al. 2015) and kernel density estimation (Ray and Reich 2018). Recent work focussed on the dependency of population flow and the viral spread (Colizza et al. 2006; Balcan et al. 2009; Belik et al. 2011; Brockmann and Helbing 2013). As shown by (Pei et al. 2018), the spread of influenza can be more accurately predicted by taking the population flow between cities into account. Read et al. (2020) predicted the COVID-19 epidemic by using the Official Aviation Guide (OAG) Traffic Analyser dataset. Additionally to the OAG dataset, (Wu et al. 2020) used the Tencent database to predict the COVID-19 viral spread.

The population flow clearly has an impact on the evolution of an epidemic. However, the exact population flow is unknown, and epidemic prediction methods must account for inaccuracies of population flow data. In this work, we consider the most extreme case by assuming no prior knowledge of the population flow. To forecast the COVID-19 epidemic, we design the network-based prediction method NIPA that estimates the interactions between cities as an intermediate step. On February 14th, 2020, approximately 75% of the global COVID-19 infections are located in the Chinese province Hubei. Thus, we focus on the COVID-19 epidemic in Hubei. More precisely, our goal is to predict the COVID-19 outbreak for every city in Hubei.

## Materials and methods

### Data on the COVID-19 epidemic outbreak in Hubei

**Fig. 1:**
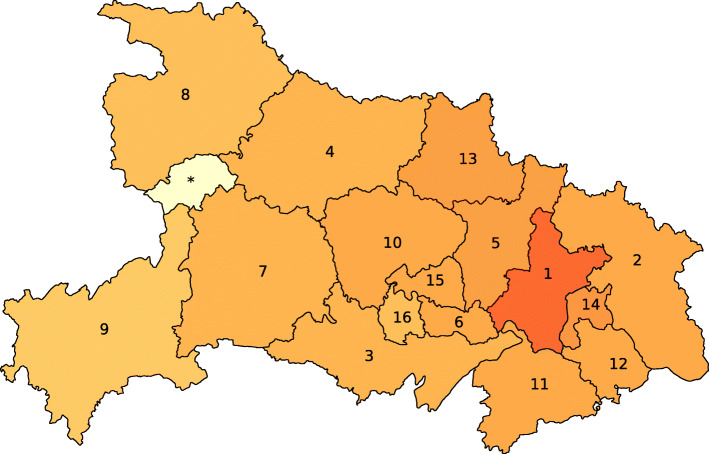
**Map of cities in Hubei**. The 17 cities (prefecture-level divisions) of the Chinese province Hubei. The names of the cities are stated in Supplementary Table S2. The greater the fraction of people that have been infected by COVID-19 on February 14, the darker the city. We do not consider the city Shennongjia in this work, which is marked with a star (*) and a light colour

The time series of reported infections in Hubei forms the basis for the epidemic outbreak prediction. Hubei is divided into 17 cities (more precisely, prefecture-level divisions) and contains the city Wuhan, as illustrated by Fig. 1. We do not consider the city Shennongjia, since the number of infections in Shennongjia is small. We denote the number of considered cities by *N*=16. The number of newly reported infections for each city in Hubei is openly accessible via the website of the Hubei Province Health Committee (http://www.hubei.gov.cn/, unpublished). The data is updated daily and follows the standard time offset of UTC+08:00. Except for Wuhan, the total number of reported infections is small before January 21, 2020. Hence, we consider the COVID-19 epidemic outbreak starting from January 21. From February 13 on, a new diagnosing method on the basis of chest scans has been used for reporting the infections in Hubei (‘Coronavirus Latest: China’s Epicentre Records No New Cases’, www.nature.com/articles/d41586-020-00154-w, unpublished). The new diagnosing method resulted in an erratic spike in the number of reported infections. We focus on predicting the number of infections of the initial diagnosing method, which is based on genetic tests. The number of reported infections of the initial diagnosing method is accessible from (http://www.hubei.gov.cn/, unpublished) until February 14, 2020. Thus, we focus on the COVID-19 epidemic in Hubei from January 21 until February 14, 2020.

We denote the discrete time by $k\in \mathbb {N}$. The difference of time *k* to *k*+1 equals one day, and the initial time *k*=1 corresponds to January 21, 2020. The website (http://www.hubei.gov.cn/, unpublished) states the number of reported infections *N*_*rep*,*i*_[*k*] at every time *k* in every city *i*=1,...,*N*. We obtain the population size *p*_*i*_ of each city *i* from the Hubei Statistical Yearbook (Li and Xu 2016). The reported fraction of infected individuals in city *i* at time *k* follows as

1 $$ \mathcal{I}_{{rep}, i}[k] = N_{{rep}, i}[k]/p_{i}.  $$

Supplementary Table S2 states the population size *p*_*i*_ and the complete time series of the number of infections *N*_*rep*,*i*_[*k*] for each city in Hubei.

### Modelling the COVID-19 epidemic between cities

We model the spread of the COVID-19 virus by the SIR-model: At any discrete time *k*, every individual is in either one of the compartments *susceptible* (healthy), *infectious* or *removed*. Susceptible individuals can get infectious due to contact with infectious individuals. Due to curing, hospitalisation, quarantine measures or death, infectious individuals become removed individuals, which cannot infect susceptible individuals any longer. For every city *i*, we denote the 3×1*viral state* vector at time *k* by

2 $$ v_{i}[k] = \left(\begin{array}{ccc} \mathcal{S}_{i}[k]\\ \mathcal{I}_{i}[k] \\ \mathcal{R}_{i}[k] \end{array}\right).  $$

The components $\mathcal {S}_{i}[k], \mathcal {I}_{i}[k]$, and $\mathcal {R}_{i}[k]$ denote the fraction of susceptible, infectious, and removed individuals, respectively. Thus, it holds that $\mathcal {S}_{i}[k]+\mathcal {I}_{i}[k]+\mathcal {R}_{i}[k] = 1$ for every city *i* at every time *k*. The discrete-time SIR model follows from applying Euler’s method to the continuous-time mean-field SIR model of (Youssef and Scoglio 2011):

#### **Definition 1**

(SIR Epidemic Model (Youssef and Scoglio 2011; Prasse and Van Mieghem 2020)) For every city *i*, the viral state $v_{i}[k] = (\mathcal {S}_{i}[k], \mathcal {I}_{i}[k], \mathcal {R}_{i}[k])^{T}$ evolves in discrete time *k*=1,2,... according to

3 $$\begin{array}{*{20}l}  \mathcal{I}_{i}[ k + 1] &= (1 - \delta_{i}) \mathcal{I}_{i}[k] + \left(1 - \mathcal{I}_{i}[k] - \mathcal{R}_{i}[k]\right) \sum\limits^{N}_{j=1} \beta_{ij} \mathcal{I}_{j}[k], \\ \mathcal{R}_{i}[ k + 1] &= \mathcal{R}_{i}[k] + \delta_{i} \mathcal{I}_{i}[k],  \end{array} $$

and the fraction of susceptible individuals follows as

$$\begin{array}{*{20}l} \mathcal{S}_{i}[k] = 1 -\mathcal{I}_{i}[k] - \mathcal{R}_{i}[k]. \end{array} $$

Here, *β*_*ij*_ denotes the *infection probability* from city *j* to city *i*, and *δ*_*i*_ denotes the *curing probability* of city *i*.

The SIR model (3) assumes that the spreading parameters *δ*_*i*_,*β*_*ij*_ do not change over time *k*. The curing probability *δ*_*i*_ quantifies the capacity of individuals in city *i* to cure from the virus. The infection probability *β*_*ij*_ specifies the number of contacts of individuals in city *j* with individuals in city *i*. We emphasise that *β*_*ii*_≠0 since individuals within one city *i* do interact with each other. The *contact network* between cities in Hubei is given by the *N*×*N* matrix

$$\begin{array}{*{20}l} B=\left(\begin{array}{cccc} \beta_{11} & \beta_{12}&... & \beta_{1N}\\ \vdots & \vdots & \ddots & \vdots\\ \beta_{N1} & \beta_{N2}&... & \beta_{NN} \end{array}\right), \end{array} $$

whose elements are probabilities 0≤*β*_*ij*_≤1. Neither the curing probabilities *δ*_*i*_ nor the infection probabilities *β*_*ij*_ are known for the COVID-19 epidemic. Potentially, it is possible to state bounds or estimates for the spreading parameters *δ*_*i*_ and *β*_*ij*_ by making use of the people flow or geographical distances between the respective cities. Nevertheless, there would remain an uncertainty regarding the precise value of the spreading parameters *δ*_*i*_ and *β*_*ij*_. In this work, we consider the most extreme case: there is no a priori knowledge on the curing probabilities *δ*_*i*_ nor the infection probabilities *β*_*ij*_.

### Network-inference-based prediction algorithm (NIPA)

We propose the NIPA method to predict the outbreak of COVID-19 virus, which consists of three steps. First, we preprocess the raw data of the confirmed number of infected individuals to obtain an SIR time series *v*_*i*_[1],...,*v*_*i*_[*n*] of the viral state for every city *i*. Here, the number of observations is denoted by *n*. Second, based on the time series *v*_*i*_[1],*v*_*i*_[2],..., we obtain estimates $\hat {\delta }_{i}$ and $\hat {\beta }_{ij}$ of the unknown spreading parameters *δ*_*i*_ and *β*_*ij*_. Third, the estimates $\hat {\delta }_{i}$ and $\hat {\beta }_{ij}$ result in an SIR model (3), which we iterate for future times *k* to predict the evolution of the 2019-Cov virus. In the following, we give an outline of the first two steps of the prediction method. We refer the reader to Supplementary Information S1 for further details on NIPA.

#### Data preprocessing

We denote the number of observations by *n*, which equals the number of days since January 21, 2020. Based on the reported number of infections *N*_*rep*,*i*_[*k*], our goal is to obtain an SIR viral state vector $v_{i}[k]= (\mathcal {S}_{i}[k], \mathcal {I}_{i}[k], \mathcal {R}_{i}[k])^{T}$ for every city *i* at any time *k*=1,...,*n*. The fraction of susceptible individuals follows as $\mathcal {S}_{i}[k] = 1 - \mathcal {I}_{i}[k] - \mathcal {R}_{i}[k]$ at any time *k*≥1. Thus, it suffices to determine the fraction of infectious individuals $\mathcal {I}_{i}[k]$ and recovered individuals $\mathcal {R}_{i}[k]$.

The fraction of infectious individuals $\mathcal {I}_{i}[k]$ follows from the reported fraction of infections $\mathcal {I}_{{rep}, i}[k]$. To be precise, the reported data is the number *N*_*rep*,*i*_[*k*] of individuals that are *detected* to be infected by COVID-19. Upon detection of the infection, the respective individuals are hospitalised and, hence, not infectious any more to individuals outside of the hospital. We consider the reported fraction of infections $\mathcal {I}_{{rep}, i}[k]$ as an *approximation* for the number of infectious individuals $\mathcal {I}_{i}[k]$. In fact, the reported fraction of infections $\mathcal {I}_{{rep}, i}[k]$ lower-bounds the true fraction of infected individuals $\mathcal {I}_{i}[k]$ for two reasons. First, not all infectious individuals are aware that they are infected. Second, the diagnosing capacities in the hospitals are limited, particularly when the number of infections increases rapidly. Hence, not all infectious individuals that arrive at a hospital can be reported timely.

We do not know the fraction of removed individuals $\mathcal {R}_{i}[k]$. At the initial time *k*=1, it is realistic to assume that $\mathcal {R}_{i}[1]=0$ holds for every city *i*. At any time *k*≥2, the removed individuals $\mathcal {R}_{i}[k]$ could be obtained from (3), if the curing probability *δ*_*i*_ were known. However, we do not know the curing probability *δ*_*i*_. Hence, we consider 50 equidistant *candidate values* for the curing probability *δ*_*i*_, ranging from *δ*_*min*_=0.01 to *δ*_*max*_=1. We define the set of candidate values as *Ω*={*δ*_*min*_,...,*δ*_*max*_}. For every candidate value *δ*_*i*_∈*Ω*, the fraction of removed individuals $\mathcal {R}_{i}[k]$ follows from (3) at all times *k*≥2. Thus, we obtain 50 potential sequences $\mathcal {R}_{i}[1],...,\mathcal {R}_{i}[n]$, each of which corresponding to one candidate value *δ*_*i*_∈*Ω*. We estimate the curing probability *δ*_*i*_, and hence implicitly the sequence $\mathcal {R}_{i}[1],...,\mathcal {R}_{i}[n]$, as the element in *Ω* that resulted in the best fit of the SIR model (3) to the reported number of infections.

The raw time series $\mathcal {I}_{{rep},i}[1],..., \mathcal {I}_{{rep},i}[n]$ exhibits erratic fluctuations. There is a single outlier in city *i*=1 (Wuhan) at time *k*=8 (January 28, 2020), which we replace by $\mathcal {I}_{{rep},1}[8]= (\mathcal {I}_{{rep},1}[7]+\mathcal {I}_{{rep},1}[9])/2$. (Potentially, the outlier is due to the increase in the maximum number of individuals that can be diagnosed in Wuhan, from 200 to 2000 individuals per day as of January 27th (https://m.chinanews.com/wap/detail/zw/sh/2020/01-28/9071697.shtml, unpublished). To reduce the fluctuations, we apply a moving average, provided by the Matlab command smoothdata, to the time series $\mathcal {I}_{{rep},i}[1],..., \mathcal {I}_{{rep},i}[n]$ of every city *i*. The preprocessed time series $\mathcal {I}_{i}[1],..., \mathcal {I}_{i}[n]$ equals the output of smoothdata.

#### Network inference

For every city *i*, the curing probability *δ*_*i*_ is estimated as one of the candidate values in the set *Ω*, as outlined above. The remaining task is to estimate the infection probabilities *β*_*ij*_. The goal of *network inference* (Peixoto 2019; Ma et al. 2019; Di Lauro et al. 2019; Timme and Casadiego 2014; Wang et al. 2016) is to estimate the matrix *B* of infection probabilities from the SIR viral state observations *v*_*i*_[1],...,*v*_*i*_[*n*]. The matrix *B* can be interpreted as a weighted adjacency matrix. We adapt a network inference approach (Prasse and Van Mieghem 2018; 2020), which is based on formulating a set of linear equations and the *least absolute shrinkage and selection operator* (LASSO) (Tibshirani 1996; Hastie et al. 2015). We remark that the network inference approach (Prasse and Van Mieghem 2020) is also applicable to general compartmental epidemic models (Sahneh et al. 2013), such as the Susceptible-Exposed-Infected-Removed (SEIR) epidemic model. The crucial observation from the SIR governing equations (3) is that *β*_*ij*_ appears linearly, whereas the state variables $\mathcal {S}_{i}, \mathcal {I}_{i}$ and $\mathcal {R}_{i}$ do not. From (3), the infection probabilities *β*_*ij*_ satisfy

4 $$\begin{array}{*{20}l} V_{i} = F_{i} \left(\begin{array}{ccc} \beta_{i1} \\ \vdots \\ \beta_{iN} \end{array}\right) \end{array} $$

for all cities *i*=1,...,*N*. Here, the (*n*−1)×1 vector *V*_*i*_ and the (*n*−1)×*N* matrix *F*_*i*_ are given by

5 $$\begin{array}{*{20}l} V_{i} = \left(\begin{array}{ccc} \mathcal{I}_{i}[2] - (1 - \delta_{i})\mathcal{I}_{i}[1]\\ \vdots \\ \mathcal{I}_{i}[n] - (1 - \delta_{i})\mathcal{I}_{i}[n-1] \end{array}\right) \end{array} $$

and

6 $$\begin{array}{*{20}l} F_{i} = \left(\begin{array}{ccc} \mathcal{S}_{i}[1] \mathcal{I}_{1}[1]&... & \mathcal{S}_{i}[1] \mathcal{I}_{N}[1] \\ \vdots & \ddots & \vdots\\ \mathcal{S}_{i}[n-1] \mathcal{I}_{1}[n-1]&... & \mathcal{S}_{i}[n-1] \mathcal{I}_{N}[n-1] \end{array}\right). \end{array} $$

If the SIR model (3) were an exact description of the evolution of the coronavirus, then the linear system (4) would hold with equality. However, the viral state vector *v*_*i*_[*k*] in city *i* does not exactly follow the SIR model (3). Instead, the evolution of the viral state vector *v*_*i*_[*k*] is described by

$$\begin{array}{*{20}l} v_{i} [k + 1] & = f_{\textrm{SIR}}(v_{1}[k],..., v_{N}[k]) + w_{i}[k], \end{array} $$

where the 3×1 vector *f*_SIR_(*v*_1_[*k*],...,*v*_*N*_[*k*]) denotes the right-hand sides of the SIR model (3), and the 3×1 vector *w*_*i*_[*k*] denotes the unknown *model error* of city *i* at time *k*. Due to the model errors *w*_*i*_[*k*], the linear system (4) only holds approximately. Thus, we resort to estimating the infection probabilities *β*_*ij*_ by minimising the deviation of the left side and the right side of (4). We infer the network by the LASSO (Tibshirani 1996; Hastie et al. 2015) as follows:

7 $$\begin{array}{*{20}l} \begin{aligned} & \underset{\beta_{i1},..., \beta_{iN}}{\operatorname{min}} & & \left\lVert V_{i} - F_{i} \left(\begin{array}{ccc} \beta_{i1} \\ \vdots \\ \beta_{iN} \end{array}\right) \right\rVert^{2}_{2} + \rho_{i} \sum\limits^{N}_{j=1, j\neq i}\beta_{ij} & \\ &{s.t.} & & 0\le \beta_{ij} \le 1, \quad j=1,..., N. &\end{aligned} \end{array} $$

The first term in the objective function of (7) measures the deviation of the left side and the right side of (4). The sum in the objective of (7) is an *ℓ*_1_–norm regularisation term which avoids overfitting. We choose to not penalise the probabilities *β*_*ii*_, since we expect the infections among individuals within the same city *i* to be dominant. The regularisation parameter *ρ*_*i*_>0 is set by cross–validation. The LASSO network inference (7) allows for the incorporation of a priori knowledge of the contact network *B* by adding further constraints to the infection probabilities *β*_*ij*_. We emphasise that an accurate prediction of an SIR epidemic outbreak does not require an accurate network inference (Prasse and Van Mieghem 2020), see also Supplementary Information S1. If the observed viral state sequence *v*_*i*_[1],..., *v*_*i*_[*n*] is generated by the SIR model (3), then NIPA accurately predicts the infection state $\mathcal {I}_{i}[k]$. Furthermore, NIPA provides accurate short-term predictions, also when the viral state *v*_*i*_[*k*] does not exactly follow the SIR model (3), i.e., in the presence of model errors *w*_*i*_[*k*]. We refer the reader to Supplementary Information S1 for further details on NIPA.

### Logistic regression

The accuracy of NIPA is evaluated by comparison to a simple prediction method. Qualitatively, the virus spread in many epidemiological models follows a sigmoid function, see also (Van Mieghem 2016). A particular sigmoid function is obtained by logistic regression. As a comparison to NIPA, we apply logistic regression on the reported fractions $\mathcal {I}_{{rep}, i}[1]$,..., $\mathcal {I}_{{rep}, i}[n]$ of infection individuals, *independently* for each city *i* in Hubei. Logistic regression is advantageous because a logistic function is a closed-form expression. Moreover, the logistic function is an approximation to the exact solution of some epidemiological models and population growth models (Verhulst 1838; Van Mieghem 2016; Prasse and Van Mieghem 2019).

A logistic curve is given by the following equation

8 $$ y(t) = \frac{y_{\infty}}{1+e^{-K(t-t_{0})}}.   $$

In our formulation, *y*(*t*) is the time-dependent fraction of infectious individuals, *t* is the time in days, where January 21 serves as initial condition (*t*=0), *y*_*∞*_ is the fraction of infected individuals when time approaches infinity, *K* is the logistic growth rate and *t*_0_ indicates the inflection point of the logistic equation. For each city in Hubei, we have applied the Matlab command lsqcurvefit to fit the reported cumulative fraction

$$\begin{array}{*{20}l} \mathcal{I}_{{rep},{cs}, i} [k] = \sum\limits^{k}_{\tau=1} \mathcal{I}_{{rep}, i} [\tau] \end{array} $$

of infected individuals to Eq (8).

## Results and discussion

**Fig. 2:**
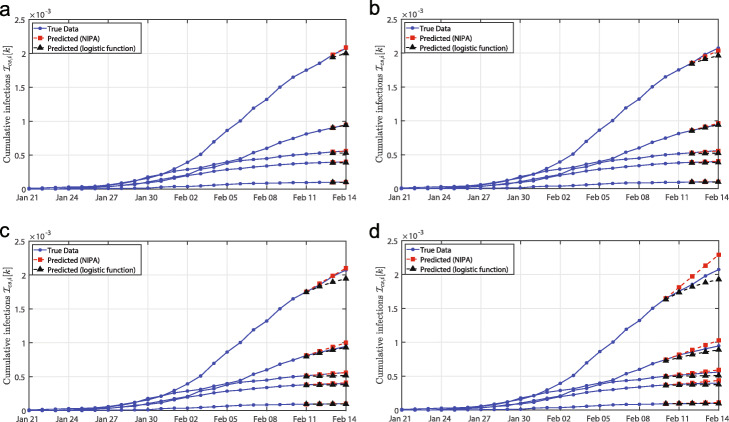
**Prediction of the COVID-19 outbreak in Hubei**. The prediction of the COVID-19 outbreak in Hubei by NIPA and by simple logistic regression. For clarity, only five of the *N*=16 cities are depicted. Each subfigure is obtained by omitting a number *m*=1,2,3,4 of days prior to February 14, 2020, and subsequently predicting the same number of days ahead in time. The omitted number of data points is equal to: **a***m*=1 day, **b***m*=2 days, **c***m*=3 days and **d***m*=4 days. The first prediction data point, for instance February 13 in subfigure **a**), coincides with the last day that has been observed

To evaluate the prediction accuracy, we remove the data for a fixed number of days, say *m*, prior to February 14. The prediction model is determined by the observation from 21 January up to 14−*m* February, 2020. Then, we predict the course of the disease up to February 14. The course of the disease is shown in Fig. 2 for the removal of *m*=1,2,3,4 days. For most predictions shown in Fig. 2, the logistic curve appears to underestimate the true fraction of infected individuals, whereas NIPA seems to overestimate the true value.

We quantify the prediction accuracy by the Mean Absolute Percentage Error (MAPE)

$$ e[k] = \frac{1}{N} \sum\limits_{i=1}^{N} \frac{\left|\hat{\mathcal{I}}_{{cs}, i}[k] - \mathcal{I}_{{cs}, i} [k]\right|}{\mathcal{I}_{{cs}, i} [k]},   $$

at any prediction time *k*≥*n*+1. Here, the predicted cumulative fraction of individuals of city *i* at time *k* equals

9 $$ \hat{\mathcal{I}}_{{cs}, i}[k] = \sum\limits^{k}_{\tau=1} \hat{\mathcal{I}}_{i} [\tau].  $$

**Fig. 3:**
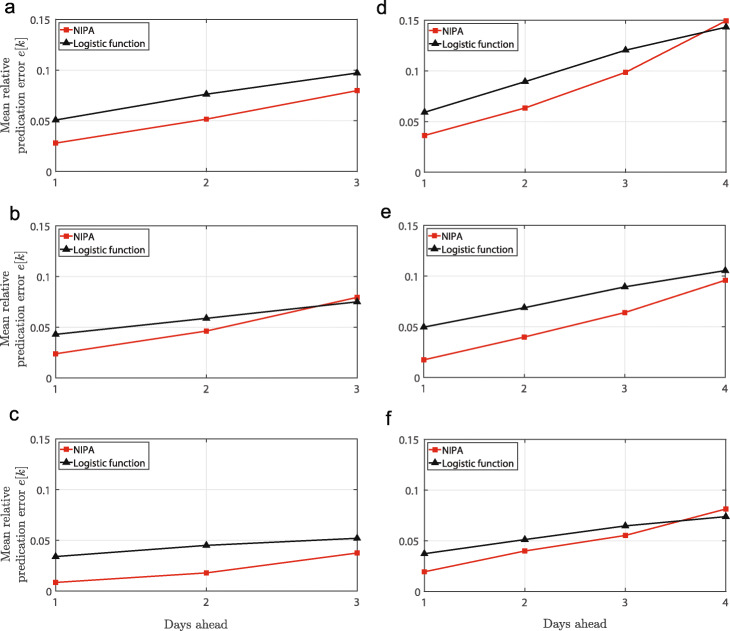
**Prediction error versus prediction time**. The prediction accuracy of NIPA and logistic regression to forecast the COVID-19 outbreak in Hubei. Each subfigure is obtained by omitting a number of days prior to February *r* and subsequently predicting the same number of days ahead in time. The subfigures of the three rows (**a**, **d**), (**b**, **e**) and (**c**, **f**) correspond to February *r*=10,*r*=12 and *r*=14, respectively. The subfigures of the two columns (**a**, **b**, **c**) and (**d**, **e**, **f**) correspond to *m*=3 and *m*=4 omitted days before February *r*, respectively

Figure 3 depicts the MAPE prediction error for the data shown in Fig. 2. Two observations are worth mentioning. First, as expected, the prediction error increases when predicting more days ahead. Second, the prediction accuracy of NIPA is almost always better than the logistic regression. In particular, NIPA provides more accurate *short-term* predictions.

**Fig. 4:**
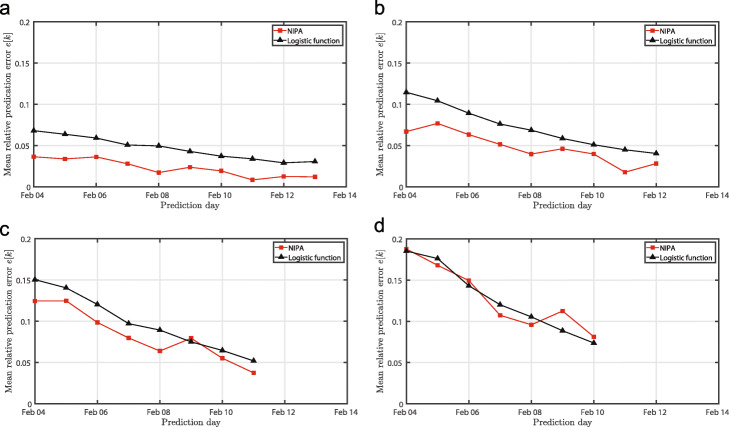
**Prediction error versus number of observed days**. The accuracy of both prediction methods for the COVID-19 outbreak versus the date until the data is available. The subfigures correspond to a prediction of : (**a**) 1-day ahead, (**b**) 2-days ahead, (**c**) 3-days ahead, (**d**) 4-days ahead

Lastly, Fig. 4 illustrates the prediction accuracy versus the time that the epidemic outbreak has been observed. As the epidemic evolves over time, the prediction accuracy of both methods increases. For nearly all forecasts, the NIPA method outperforms logistic regression. Also, as expected, forecasting more days ahead always decreases the prediction accuracy for both prediction methods.

## Conclusion

We applied a network-based SIR epidemic model to predict the outbreak of the COVID-19 virus for each city in the Chinese province Hubei. The epidemic model allows to explicitly specify the interactions of individuals of different cities, for instance by using traffic patterns between cities. However, the precise interactions between cities is unknown and must be inferred from observing the evolution of the epidemic.

We proposed the NIPA prediction method, which estimates the interactions between cities as an intermediate step. We did not assume any prior knowledge on the interactions between cities. The prediction method is evaluated on past data of the COVID-19 outbreak in Hubei. Our results indicate that a network-based modelling approach may yield more accurate predictions than modelling the epidemic for each city independently. We believe that the prediction accuracy of NIPA could be further improved, e.g., by using traffic flow patterns as prior knowledge.

## Supplementary information

**Additional file 1** Appendix S1 – Details of NIPA. The details and pseudocode of the Network-Inference-based Prediction Algorithm (NIPA). Furthermore, the prediction accuracy of NIPA is evaluated on the SIR epidemic model.

**Additional file 2** Table S2 – Data of the COVID-19 epidemic outbreak in Hubei. The time series of the reported number of infections and the population size for every city in Hubei.

## Data Availability

All data generated or analysed during this study are included in this published article [and its supplementary information files].

## Abbreviations

COVID-19: Coronavirus disease 2019
LASSO: Least absolute shrinkage and selection operator
MAPE: Mean absolute percentage error
NIPA: Network inference-based prediction algorithm
OAG: Official aviation guide
SARS-CoV-2: severe acute respiratory syndrome coronavirus 2
SIR: Susceptible infected removed (epidemic model)
