# Supplementary material for: Network-inference-based prediction of the COVID-19 epidemic outbreak in the Chinese province Hubei
Source: Appl Netw Sci. 2020 Jul 8;5(1):35. doi: 10.1007/s41109-020-00274-2 (PMC7341469; doi:10.1007/s41109-020-00274-2)
Supplement: Supplementary file 1 — Additional file 1 Appendix S1 – Details of NIPA. The details and pseudocode of the Network-Inference-based Prediction Algorithm (NIPA). Furthermore, the prediction accuracy of NIPA is evaluated on the SIR epidemic model. [file 41109_2020_274_MOESM1_ESM.pdf]

Appendix: Network Inference-based Prediction of the COVID-19 Outbreak in the Chinese Province Hubei

Bastian Prasse[1*], Massimo A. Achterberg[1], Long Ma[1] and Piet Van Mieghem[1]

1 Faculty of Electrical Engineering, Mathematics and Computer Science, P.O Box 5031, 2600 GA Delft, The Netherlands

* b.prasse@tudelft.nl

Constraints on the Infection Probabilities

To ensure that the fraction of infected individuals $\mathcal{I}_i[k]$ does not exceed one at every time k, we have added the second constraint in the LASSO (7). Indeed, the second constraint in the LASSO (7) ensures that $\mathcal{I}_i[k] \leq 1$ at every time k, as shown by Lemma 1, whose proof is inspired by [1].

Lemma 1. *Suppose that* $\mathcal{I}_i[1] \geq 0$, $\mathcal{R}_i[1] \geq 0$ *and* $\mathcal{I}_i[1]+\mathcal{R}_i[1] \leq 1$ *holds for every node* i. *Furthermore, assume that the curing probabilities* δ_i *satisfy* $0 \leq \delta_i \leq 1$ *and the infection probabilities* $\beta_{ij} \geq 0$ *satisfy*

$$\sum_{j=1}^{N} \beta_{ij} \leq 1. \tag{10}$$

Then, it holds that $\mathcal{I}_i[k] \geq 0$, $\mathcal{R}_i[k] \geq 0$ *and* $\mathcal{I}_i[k] + \mathcal{R}_i[k] \leq 1$ *at every time* $k \in \mathbb{N}$ *for every node* i.

Proof. We prove Lemma 1 by induction. Suppose that at time k for every node i it holds that

$$\mathcal{I}_i[k] \geq 0 \tag{11}$$

and

$$\mathcal{R}_i[k] \geq 0 \tag{12}$$

and

$$\mathcal{I}_i[k] + \mathcal{R}_i[k] \leq 1. \tag{13}$$

By assumption, it holds that $0 \leq \delta_i \leq 1$ and $\beta_{ij} \geq 0$. Thus, we obtain from the SIR governing equations (3) and (13) yield that both $\mathcal{I}_i[k+1]$ and $\mathcal{R}_i[k+1]$ equal a sum of positive addends, which implies that

$$\mathcal{I}_i[k+1] \geq 0 \tag{14}$$

and

$$\mathcal{R}_i[k+1] \geq 0. \tag{15}$$

Furthermore, we obtain for every node i that

$$\mathcal{I}_i[k+1] + \mathcal{R}_i[k+1] = \mathcal{I}_i[k] + \mathcal{R}_i[k] + (1 - \mathcal{I}_i[k] - \mathcal{R}_i[k]) \sum_{j=1}^{N} \beta_{ij} \mathcal{I}_j[k]. \tag{16}$$

From (11), (12) and (13), we obtain that $\mathcal{I}_i[k]+\mathcal{R}_i[k] \in [0,1]$. Since (12) and (13) imply that $\mathcal{I}_i[k] \leq 1$, it holds that

$$\sum_{j=1}^{N} \beta_{ij} \mathcal{I}_j[k] \leq \sum_{j=1}^{N} \beta_{ij} \leq 1. \tag{17}$$

Thus, $\mathcal{I}_i[k+1] + \mathcal{R}_i[k+1] \leq 1$, since the right side of (16) is a convex combination of 1 and $\sum_{j=1}^{N} \beta_{ij} \mathcal{I}_j[k] \in [0,1]$. □

Details of NIPA

Algorithm 1 describes the NIPA prediction method in pseudocode[1]. In line 4, the Matlab command `smoothdata` is called to remove erratic fluctuations of the raw data $\mathcal{I}_{\text{rep},i}[k]$. We denote the $N \times 1$ infection state vector by $\mathcal{I}[k] = (\mathcal{I}_1[k], ..., \mathcal{I}_N[k])^T$ at any time k. The loop starting in line 8 iterates over all candidate values of the curing probability δ_i which are in the set Ω. Algorithm 1 calls the network inference method, which is stated in pseudocode by Algorithm 2. For a fixed curing probability δ_i, the network inference in line 12 returns an estimate for the infection probabilities $\beta_{i1}(\delta_i)$, ..., $\beta_{iN}(\delta_i)$. Furthermore, the network inference returns the mean squared error $\text{MSE}(\delta_i)$, which corresponds to the first term in the objective of (7). The smaller the mean squared error $\text{MSE}(\delta_i)$, the better the fit of the SIR model (3) to the data $\mathcal{I}_i[1], ..., \mathcal{I}_i[n]$. In line 14, the final estimate $\hat{\delta}_i$ for the curing probability is obtained as the minimiser of the mean squared error $\text{MSE}(\delta_i)$. The estimate $\hat{\delta}_i$ determines the final estimates $\hat{\beta}_{i1}$, ..., $\hat{\beta}_{iN}$ for the infection probabilities in line 15. From line 17 to line 27, the SIR model (3) is iterated, which results in the predicted fraction of infections $\hat{\mathcal{I}}_i[n+1], ..., \hat{\mathcal{I}}_i[n+n_{\text{pred}}]$ for all cities i.

To determine the regularisation parameter ρ_i in the LASSO (7), we consider 100 candidate values, specified by the set $\Theta_i = \{\rho_{\min,i}, ..., \rho_{\max,i}\}$. In line 4 of Algorithm 2, the maximum value is set to $\rho_{\max,i} = 2\|F_i^T V_i\|_\infty$. If $\rho_i > \rho_{\max,i}$, then [2] the solution to the LASSO (7) is $\beta_{ij} = 0$ for all cities j. For every value of the regularisation parameter $\rho_i \in \Theta_i$, we compute the mean squared error $\text{MSE}(\delta_i, \rho_i)$ by 3-fold cross–validation [3]. For every fold, the rows of the matrix F_i and the vector V_i are divided into a training set $F_{i,\text{train}}$, $V_{i,\text{train}}$ and a validation set $F_{i,\text{val}}$, $V_{i,\text{val}}$. We compute the solution β_{i1}, ..., β_{iN} to the LASSO (7) on the training set of every fold $F_{i,\text{train}}$, $V_{i,\text{train}}$. The mean squared error $\text{MSE}(\delta_i, \rho_i)$ then equals

$$\left\| V_{i,\text{val}} - F_{i,\text{val}} \begin{pmatrix} \beta_{i1} \\ \vdots \\ \beta_{iN} \end{pmatrix} \right\|_2^2,$$

averaged over all folds. Finally, we set the regularisation parameter ρ_i to the minimiser of $\text{MSE}(\delta_i, \rho_i)$. The final estimate $\beta_{i1}(\delta_i), ..., \beta_{iN}(\delta_i)$ for the infection probabilities is obtained by solving the LASSO (7)

[1] The Matlab code is available upon request to the authors.

Algorithm 1 `Network Inference-based Prediction Algorithm (NIPA)`

Input: reported fraction of infections $\mathcal{I}_{\text{rep},i}[1], ..., \mathcal{I}_{\text{rep},i}[n]$ for all cities i; prediction time n_{pred}
Output: predicted fraction of infections $\hat{\mathcal{I}}_i[n+1], ..., \hat{\mathcal{I}}_i[n+n_{\text{pred}}]$ for all cities i

Step 1 – Data preprocessing

$\mathcal{I}_{\text{rep},1}[17] \leftarrow (\mathcal{I}_{\text{rep},1}[16] + \mathcal{I}_{\text{rep},1}[18])/2$
$\mathcal{I}_i[1], ..., \mathcal{I}_i[n] \leftarrow$ `smoothdata`$(\mathcal{I}_{\text{rep},i}[1], ..., \mathcal{I}_{\text{rep},i}[n])$ for all $i = 1, ..., N$
$\mathcal{I}[k] \leftarrow (\mathcal{I}_1[k], ..., \mathcal{I}_N[k])^T$ for all $k = 1, ..., n$

Step 2 – Network inference

for $i = 1, ..., N$ **do**
    $\mathcal{R}_i[1] \leftarrow 0$
    for $\delta_i \in \Omega$ **do**
        $\mathcal{R}_i[k] \leftarrow \mathcal{R}_i[k-1] + \delta_i \mathcal{I}_i[k-1]$ for all $k = 2, ..., n$
        $\mathcal{S}_i[k] \leftarrow 1 - \mathcal{I}_i[k] - \mathcal{R}_i[k]$ for all $k = 1, ..., n$
        $v_i[k] \leftarrow (\mathcal{S}_i[k], \mathcal{I}_i[k], \mathcal{R}_i[k])^T$ for all $k = 1, ..., n$
        $(\beta_{i1}(\delta_i), ..., \beta_{iN}(\delta_i), \text{MSE}(\delta_i)) \leftarrow$ `Network inference`$(\delta_i, v_i[1], ..., v_i[n], \mathcal{I}[1], ..., \mathcal{I}[n])$
    end for
    $\hat{\delta}_i \leftarrow \underset{\delta_i \in \Omega}{\text{argmin}}\, \text{MSE}(\delta_i)$
    $(\hat{\beta}_{i1}, ..., \hat{\beta}_{iN}) \leftarrow \beta_{i1}(\hat{\delta}_i), ..., \beta_{iN}(\hat{\delta}_i)$
end for

Step 3 – Iterating SIR model

for $i = 1, ..., N$ **do**
    $\hat{\mathcal{I}}_i[n] \leftarrow \mathcal{I}_i[n]$
    $\hat{\mathcal{R}}_i[1] \leftarrow 0$
    $\hat{\mathcal{R}}_i[k] \leftarrow \hat{\mathcal{R}}_i[k-1] + \hat{\delta}_i \mathcal{I}_i[k-1]$ for all $k = 2, ..., n$
end for
for $k = n+1, ..., n+n_{\text{pred}}$ **do**
    for $i = 1, ..., N$ **do**
        $\hat{\mathcal{I}}_i[k] \leftarrow (1 - \hat{\delta}_i)\hat{\mathcal{I}}_i[k-1] + (1 - \hat{\mathcal{I}}_i[k-1] - \hat{\mathcal{R}}_i[k-1]) \sum_{j=1}^{N} \hat{\beta}_{ij} \hat{\mathcal{I}}_j[k-1]$
        $\hat{\mathcal{R}}_i[k] \leftarrow \hat{\mathcal{R}}_i[k-1] + \hat{\delta}_i \hat{\mathcal{I}}_i[k-1]$
    end for
end for

on the whole matrix F_i and vector V_i. To solve the LASSO (7) numerically, we make use of the Matlab command `quadprog`.

Algorithm 2 `Network inference`

Input: curing probability δ_i; viral state $v_i[k]$ for $k = 1, ..., n$; infection state vector $\mathcal{I}[k]$ for $k = 1, ..., n$
Output: infection probability estimates $\beta_{i1}(\delta_i), ..., \beta_{iN}(\delta_i)$; mean squared error $\text{MSE}(\delta_i)$
Compute V_i and F_i by (5) and (6)
$\rho_{\max,i} \leftarrow 2\|F_i^T V_i\|_\infty$
$\rho_{\min,i} \leftarrow 10^{-4}\rho_{\max,i}$
$\Theta_i \leftarrow$ 100 logarithmically equidistant values from $\rho_{\min,i}$ to $\rho_{\max,i}$
for $\rho_i \in \Theta_i$ **do**
    estimate $\text{MSE}(\delta_i, \rho_i)$ by 3-fold cross–validation on F_i, V_i and solving (7) on the respective training set
end for
$\rho_{\text{opt},i} \leftarrow \underset{\rho_i \in \Theta_i}{\text{argmin}}\, \text{MSE}\,(\delta_i, \rho_i)$
$(\beta_{i1}(\delta_i), ..., \beta_{iN}(\delta_i)) \leftarrow$ the solution to (7) on the whole data set F_i, V_i for $\rho_i = \rho_{\text{opt},i}$
$\text{MSE}(\delta_i) \leftarrow \text{MSE}(\delta_i, \rho_{\text{opt},i})$

Evaluation of the Prediction Accuracy of NIPA

We generate viral state sequences $v_i[1]$, ...,$v_i[n]$ according to the SIR model (3) to evaluate the prediction accuracy of NIPA. We set the number of nodes to $N = 16$ and the number of observations to $n = 20$. The curing probabilities δ_i are set to a random number, uniformly distributed in $[0.5, 1]$. We perform two steps to generate the contact network B. First, we generate an $N \times N$ adjacency matrix A with zero-one elements $a_{ij} \in \{0, 1\}$ based on the (directed) Erdős-Rényi graph model [4]. For any two nodes $i \neq j$, we set $a_{ij} = 1$ with link probability $p = 0.3$. If the resulting graph is not connected, then we repeat the random graph generation. We set the diagonal elements to $a_{ii} = 1$ for every node i. Second, we set the elements of the matrix B to $\beta_{ij} = 0$ if $a_{ij} = 0$. If $a_{ij} = 1$, then we set the infection probability β_{ij} to a random number, uniformly distributed in $[0.1, 0.2]$.

Fig 1 shows that NIPA accurately predicts the infection state $\mathcal{I}_i[k]$ for every node i at every time $k \geq n + 1$. We emphasise that the true curing rates δ_i are not in the set Ω (with probability one). Thus, the estimates $\hat{\delta}_i$ of NIPA cannot exactly equal the true curing rates δ_i.

We denote the estimated contact network with the elements $\hat{\beta}_{ij}$ by $\hat{B}$. To assess whether the estimated matrix $\hat{B}$ is similar to the true matrix B, we calculate the area under the receiver-operating-characteristic curve (AUC) [5]. The AUC lies between 0 and 1, and an AUC of 1/2 is equivalent to tossing a coin to determine the presence ($\hat{\beta}_{ij} = 1$) or absence ($\hat{\beta}_{ij} = 0$) of a link. If the inferred network $\hat{B}$ equals the true network B, then the AUC equals 1. The AUC of the estimate $\hat{B}$ of Fig 1 equals merely 0.53. Hence, the topology of the estimate $\hat{B}$ and the true matrix B have virtually no resemblance, which is in agreement with [6].

We perform further simulations to evaluate the robustness of NIPA against model errors $w_i[k]$. We

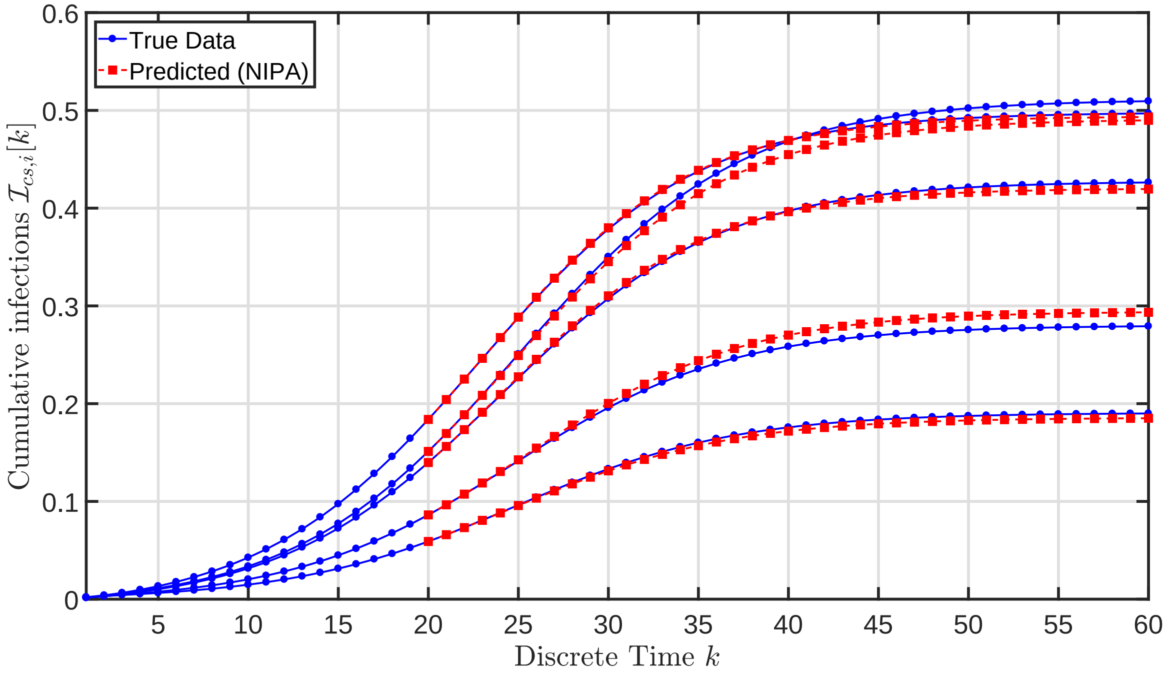

Figure 1: **NIPA prediction for an exact SIR epidemic.** The true cumulative infection state $\mathcal{I}_{cs,i}[k]$ and the prediction of NIPA. The infection state $\mathcal{I}_i[k]$ has been generated by the SIR model (3). For clarity, only the infection states $\mathcal{I}_i[k]$ of five of the $N = 16$ nodes are depicted.

generate viral state sequences $v_i[1]$, ...,$v_i[n]$ similarly as in Fig 1, but we replace the infection state $\mathcal{I}_i[k]$ for every node i at every time k by

$$\mathcal{I}_i[k] \leftarrow \mathcal{I}_i[k] + w_i[k]. \tag{18}$$

Here, $w_i[k]$ denotes additive white Gaussian noise with zero mean and standard deviation $\sigma = 0.002$. The model errors $w_i[k]$ and $w_j[k]$ are stochastically independent for all nodes $i \neq j$. If the infection state $\mathcal{I}_i[k]$ resulting from (18) is negative, we set $\mathcal{I}_i[k] \leftarrow |\mathcal{I}_i[k]|$. Fig 2 demonstrates of the intensity of the model error $w_i[k]$.

Fig 3 demonstrates the NIPA prediction accuracy for the SIR model with model errors $w_i[k]$. For times k that are considerably greater than the observation time n, the prediction of the cumulative infection state $\mathcal{I}_{cs,i}[k]$ diverges from true infection state. However, the predictions of NIPA are accurate until approximately $k \approx n + 5$, which is valuable for short-term disease counter-measures.

References

[1] Paré PE, Liu J, Beck CL, Kirwan BE, Başar T. (2018) Analysis, estimation, and validation of discrete-time epidemic processes. IEEE Transactions on Control Systems Technology.

[2] Kim SJ, Koh K, Lustig M, Boyd S, Gorinevsky D. (2007) An interior-point method for large-Scale l_1-regularized least squares. IEEE journal of selected topics in signal processing 1(4):606–617.

[3] Hastie T, Tibshirani R, Wainwright M. (2015) Statistical learning with sparsity: the lasso and generalizations. CRC press.

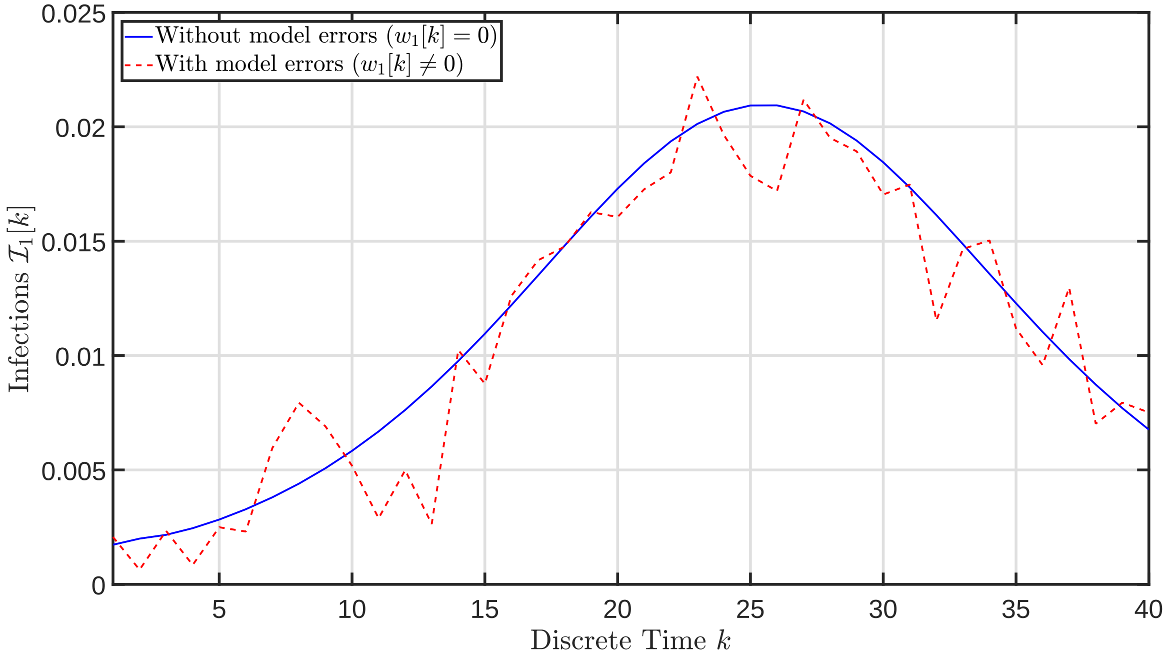

Figure 2: **SIR with and without model errors.** The SIR infection state $\mathcal{I}_1[k]$ of one node with and without normally distributed model errors $w_i[k]$ with standard deviation $\sigma = 0.002$.

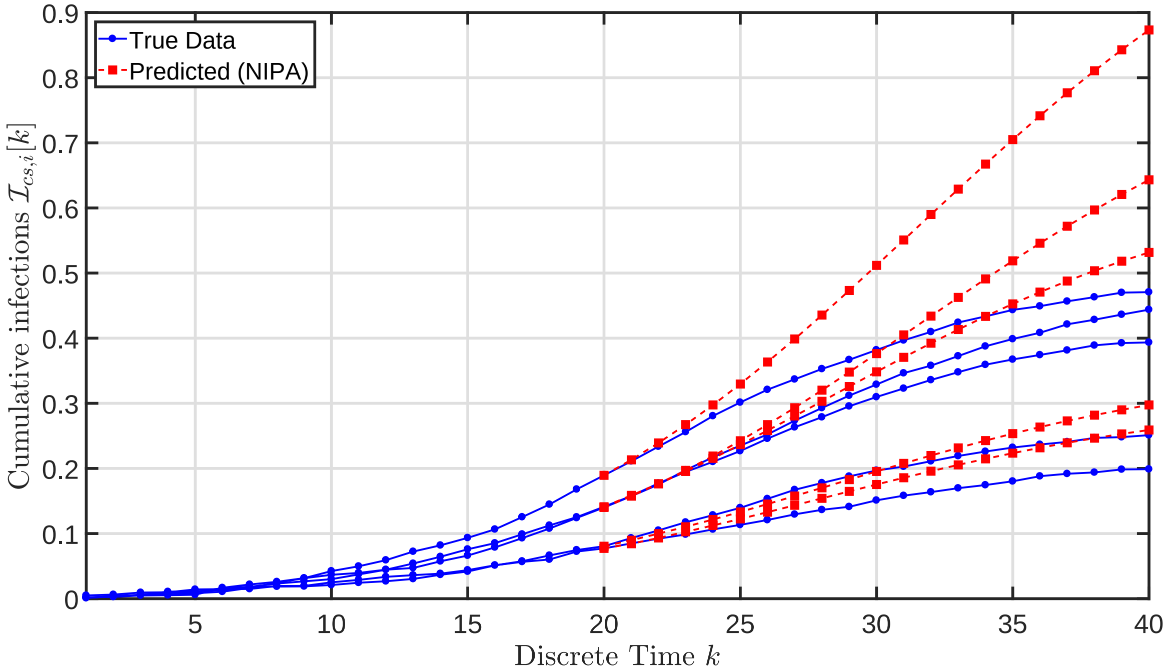

Figure 3: **NIPA prediction for an SIR epidemic with model errors.** The true cumulative infection state $\mathcal{I}_{cs,i}[k]$ and the prediction of NIPA. The infection state $\mathcal{I}_i[k]$ has been generated by the SIR model (3) plus normally distributed model errors $w_i[k]$ with standard deviation $\sigma = 0.002$. For clarity, only the infection states $\mathcal{I}_i[k]$ of five of the $N = 16$ nodes are depicted.

[4] Erdős P and Rényi A. (1959) On the evolution of random graphs. Publ. Math. Inst. Hung. Acad. Sci. 5:17–60.

Table 1: The Mean Absolute Percentage Error for subfigure 2d for the different cities.

City	1 day		2 days		3 days		4 days	
	NIPA	Logistic	NIPA	Logistic	NIPA	Logistic	NIPA	Logistic
Wuhan	0.0332	0.0096	0.0634	0.0193	0.0767	0.0497	0.1049	0.0701
Huanggang	0.0115	0.0354	0.0342	0.0424	0.0598	0.0525	0.0969	0.0576
Jingzhou	0.0121	0.0242	0.0224	0.0425	0.0340	0.0631	0.0409	0.0915
Xiangyang	0.0223	0.0485	0.0587	0.0507	0.1019	0.0530	0.1459	0.0598
Xiaogan	0.0049	0.0066	0.0177	0.0036	0.0281	0.0195	0.0565	0.0214
Xiantao	0.0170	0.0090	0.0431	0.0145	0.0636	0.0305	0.0952	0.0395
Yichang	0.0368	0.0389	0.0610	0.0596	0.1026	0.0709	0.1513	0.0806
Shiyan	0.0214	0.0483	0.0225	0.0699	0.0194	0.0938	0.0050	0.1025
Enshi	0.0320	0.0849	0.0667	0.1047	0.1042	0.1216	0.1659	0.1198
Jingmen	0.0298	0.0324	0.0358	0.0549	0.0264	0.0694	0.0057	0.0677
Xianning	0.0097	0.0045	0.0394	0.0009	0.0508	0.0189	0.0741	0.0328
Huangshi	0.0019	0.0215	0.0102	0.0291	0.0304	0.0341	0.0491	0.0459
Suizhou	0.0155	0.0333	0.0305	0.0489	0.0375	0.0739	0.0573	0.0906
Ezhou	0.0054	0.0457	0.0339	0.0460	0.0612	0.0509	0.0866	0.0604
Tianmen	0.0062	0.1392	0.0467	0.2145	0.0005	0.2151	0.0561	0.2091
Qianjiang	0.0494	0.0126	0.0516	0.0163	0.0861	0.0168	0.1092	0.0304

[5] Fawcett T. (2006) An introduction to ROC analysis. Pattern recognition letters 27(8):861–874.

[6] Prasse B, Van Mieghem P. (2020) Network reconstruction and prediction of epidemic outbreaks for general group-based compartmental epidemic models. IEEE Transactions of Network Science and Engineering.
